# Development and Validation of Chinese Parental Involvement and Support Scale for Preschool Children

**DOI:** 10.3389/fpsyg.2022.792910

**Published:** 2022-04-11

**Authors:** Yaping Yue, Xiangru Zhu, Yisi Zhang, Wanyu Ren

**Affiliations:** ^1^School of Education, Henan University, Kaifeng, China; ^2^School of Psychology, Henan University, Kaifeng, China; ^3^Sanmenxia Polytechnic, Sanmenxia, China; ^4^Kaifeng Vocational College of Culture and Arts, Kaifeng, China

**Keywords:** preschool children, parental involvement, parental support, scale development, scale validation, China

## Abstract

The present study developed the Chinese Parental Involvement and Support Scale for Preschool Children (CPISSPC) to measure parental involvement and support for preschool children. In Study 1, we conducted a literature review, open-ended interviews, a theoretical analysis, and expert interviews to create an item bank (58 items). In Study 2, 447 parents completed the item bank. Following item and Exploratory Factor Analysis, 30 items were retained. In Study 3, five new items were added to the 30-item version of the CPISSPC. A separate sample of 471 parents completed the 35-item version of the CPISSPC. After Confirmatory Factor Analysis, a final 18-item version of the CPISSPC was created with four factors: psychological support, educational support, play support, and life support. Construct validity and internal reliability (Cronbach’s α = 0.88) were satisfactory. Study 4 evaluated concurrent validity (*n* = 318). CPISSPC scores significantly and positively correlated with perceived social support, marital gratification, and children’s self-efficacy. CPISSPC scores significantly and negatively correlated with parents’ levels of burnout and loneliness. The CPISSPC exhibits good psychometric properties and can be used as a tool to measure parental involvement and support for preschool children.

## Introduction

The family has a strong influence on children’s development. From children’s first moments of life, they depend on parents and the family to protect them and provide for their needs. Parental involvement and support are very broad concepts. Different scholars have different opinions about them (Hoagwood et al., 2010; UNICEF, 2014; Chen, 2017). The present study was based on views of previous scholars, combined with definitions of parental involvement and support for preschool children. Parental involvement and support refer to life support, psychological support, educational support, and play support that are provided by the family and the main caregivers to preschool children. These forms of support have a vital impact on the growth and development of children’s psychology, emotional wellbeing, education, behavior, and social support. Research has shown that high levels of parental involvement are related to better academic performance, higher test scores, more positive attitudes about education, higher homework completion rates, fewer special education placements, academic perseverance, lower dropout rates, and fewer suspensions (Hoover-Dempsey and Sandler, 1997; Fan and Chen, 2001; Liu and Liu, 2016). However, studies of parental involvement and support have predominantly focused on school children (Blair, 2002; Dearing et al., 2006; Hill and Tyson, 2009). Parental involvement and support for preschool children have been understudied.

The early development of children is closely related to familial economic level, expectation, intervention, and the parent-child relationship (Chan and Li, 2020; McCoy et al., 2020). It is difficult for individuals to mature without familial influence. The United States Head Start program views “parent involvement” as basic service content, giving great importance to parental involvement, support, and contributions to their children’s education. It guarantees various opportunities for parents and family members to be involved in the program’s educational services and policy implementation (U.S. Department of Health and Human Services, 1997). This is similar to viewing the family as central, and the government, social organizations, and kindergartens should regard the family as an intermediary to provide support for the growth and development of preschool children. The Head Start program also regards children and parents as a whole and actively encourages parents to participate in children’s growth and development, including healthcare and education, such as taking children to regular dental examinations, providing them with a healthy diet, and creating a warm family atmosphere (Wang, 2018). Individualized parental involvement and support are important for human development. Emotional, cognitive, linguistic, physical, and social development is inseparable from the care and support of family members (Tracey and Young, 2002; Kelly and Emery, 2003; Raikes et al., 2006; Li et al., 2020).

Many researchers have proposed various dimensions of parental involvement and support, one of which is life support, which significantly impacts preschool children’s development (U.S. Department of Health and Human Services, 1997; Wang, 2018). Research indicates that family life support for young children mainly includes stable housing, convenient transportation, and sufficient play space (Wen et al., 2004; Li et al., 2019). Preschool children exhibit improvements in cognitive and social skills when they are raised with adequate material support, which better prepares them for formal education (Zhou et al., 2019). Conversely, children who are raised in families with low socioeconomic status experience more challenges, such as low self-confidence, withdrawal, and minimal social skills (McLoyd, 1998).

Another component of parental involvement and support is psychological support, which mainly refers to the quality of parent-child interactions, which can have a long-term effect on cognitive and psychosocial development (Moss et al., 1998). Parents should treat their children kindly. Communicating with preschool child is an important parental practice. Positive two-way communication is essential for building preschool children’s self-esteem (Jackson et al., 1998; Bajaj et al., 2016). Parents should trust their children to be responsible and safe within their realm of responsibility. A lack of trust can discourage children from self-exploration and experience. Feeling loved is a key determinant of children’s healthy development (Sroufe, 2005; Hoagwood et al., 2010). For preschool children, feeling loved typically derives from their parents. Children with more loving parents benefit from greater peer competence during childhood and exhibit fewer behavioral problems (Groh et al., 2014). Verbal praise is positive feedback that conveys approval (Brophy, 1981). A previous study indicated that praise can boost children’s feelings of competence and confidence (Leijten et al., 2016).

A third component of parental involvement and support is educational support (Berger, 1991; Jeynes, 2010). A preschooler’s level of intellect is not yet mature, and they usually are not encouraged to learn academic knowledge. Therefore, educational support in the present study does not refer to learning academic knowledge; instead, it refers to parents’ guiding and supporting children to interact with peers, teaching good manners, improving independence (e.g., dressing and feeding themselves), resolving conflicts with peers, and so on. Previous studies found that these aspects are important for the development of children. For example, having the ability to dress themselves is considered essential for autonomous living (Miller et al., 2011). Another study found that kindergartners who share, cooperate, and are helpful are more likely to eventually have a college degree and gainful employment 20 years later than children who lack these social skills (Jones et al., 2015).

Lastly, accessible play materials and the ways in which parents engage with their children during play have a profound impact on children’s learning and development (Bodrova and Leong, 2007). Deciding types of suitable play for children has an important positive influence on children’s play and cognitive, social, and emotional abilities (Slater, 1995; Lin and Yawkey, 2014). Research indicates that adults’ support for infant play primarily involves providing play materials, showing interest in play, demonstrating how to play, and being willing to cooperate with them. Shen (2020) found that parent-child play is helpful for children’s psychological independence and self-control. Although the concept of “parental involvement and support” was not explicitly mentioned in these cited studies, various support factors, such as parental stress, marital satisfaction, parenting style, parent-child interactions, family socioeconomic status, and parental play support have significant impacts on preschool children’s personality development, emotional development, logical thinking ability, health, and play behavior.

In the past two decades, six scales have mainly been used to explore parenting among the Chinese population. These six scales can be classified into two categories. The first category mainly measures parents’ emotional quality with children. For example, the Self-Expressiveness within the Family Questionnaire (SEFQ) measures positive and negative expressiveness toward children (Cheng et al., 2018). The Parental Acceptance and Rejection Questionnaire (PARQ) explores parents’ warmth and rejection toward children (Wang, 2016). The Parent-Child Conflict Tactics Scale (CTSPC) mainly investigates parent-child conflict (Cui et al., 2016). The second category mainly measures parenting practices. For example, the Child Rearing Practice Report (CRPR) mainly assesses child-rearing attitudes and behavior (Chen et al., 1998; Lai et al., 2000). The Parenting Practices Emphasized in China (CPPM) scale is an 18-item measure of five parenting practices: encouragement of modesty, protection, maternal involvement, shaming/love withdrawal, and directiveness (Wu et al., 2002). Ip et al. (2018) developed the eight-item Chinese Parent-Child Interaction Scale (CPCIS), which measures learning activities (three items) and recreational activities (five items). Dong et al. (2021) translated and validated the 54-item Chinese version of the Comprehensive Early Childhood Parenting Questionnaire (CECPAQ-CV), which includes five factors: support, stimulation, structure, harsh discipline, and positive discipline. Nonetheless, these existing scales have several shortcomings with regard to assessing parental involvement and support. For example, these scales generally consist of few parenting dimensions, especially the emotional dimension. Moreover, these scales are only suitable for parents with children who are younger than 4 years of age. There is currently no adequate and comprehensive Chinese assessment tool to measure parental support and involvement for younger children. The present study sought to fill this research gap.

## The Present Study

The present study developed and validated a scale to measure parental involvement and support for preschool children in China. The study had two primary research purposes. The first purpose was to develop the Chinese Parental Involvement and Support Scale for Preschool Children (CPISSPC). The second purpose was to evaluate its structural validity and reliability.

We conducted four sub-studies. In Study 1, we sought to develop initial items of the scale, check applicability and accuracy of the items, and then form the initial scale. In Study 2, we analyzed the initial scale that was formed in Study 1 and deleted items that did not meet the requirements. Effective common factors and dimensions were formed. Study 3 revised the 30 items that were formed in the last round of testing, making expressions more concise, clearer, and easier to understand. The content of factor 4 was also expanded to keep the number of items in each dimension of the scale balanced. Exploratory Factor Analysis was then performed again to explore dimensions of the scale. In Study 4, we first reordered 18 results from Study 3 for Study 4. We then used the prediction model of the scale structure to fit the actual model and performed reliability and validity analyses to verify the scale’s reliability and validity.

## Materials and Methods

### Participants and Procedures

The study was approved by the Research Ethics Committee at the authors’ institution. Written consent was obtained from the participants before the study. All participants were randomly selected among the parents of preschool children, aged 2.5–6 years, in Henan Province, China. For the interviews, we randomly selected 24 parents (16 mothers and eight fathers) and six grandparents who had lived with preschool children and their parents for a long time. We interviewed them face-to-face about their opinions of what constitutes parental support and involvement. In the following study, the scales was distributed and collected through the Internet. Web scales were selected because of their ability to reach a large number of parents from different areas. In Study 2, a total of 483 scales were distributed, and 447 were collected, with a collection rate of 92.5% (Table 1). In Study 3, 502 scales were distributed randomly, and 471 were collected, with a collection rate of 93.8% (Table 2). Study 4 had two different samples. Among Sample 1, 480 scales were distributed, and 442 valid scales were obtained, with an effective rate of 92.1% (Table 3). Among Sample 2, 350 scales were distributed, and 318 valid scales were obtained, with an effective rate of 90.9% (Table 4).

**TABLE 1:** Basic participant information in Study 2 (*n* = 447).

Participant’s information	Classification	Frequency	Percentage (%)
Family residence	Rural	199	44.5
	Township	10	2.2
	County	17	3.8
	City	221	49.4
Educational background	Primary school	13	2.9
	Junior high school	100	22.4
	High school or technical Secondary school	91	20.4
	Junior college	103	23
	Undergraduate	117	26.2
	Postgraduate and above	23	5.1
Total monthly household income (yuan)	0–2000	84	18.8
	2001–5000	196	43.8
	5001–10000	107	23.9
	10001 and above	60	13.4
Gender of preschool children	Boy	246	55
	Girl	201	45

**TABLE 2:** Basic participant information in Study 3 (*n* = 471).

Participant’s information	Classification	Frequency	Percentage (%)
Family residence	Rural	130	27.6
	Township	64	13.6
	County	4	0.8
	City	273	58
Educational background	Primary school	26	5.5
	Junior high school	94	20
	High school or technical Secondary school	76	16.1
	Junior college	95	20.2
	Undergraduate	154	32.7
	Postgraduate and above	26	5.5
Total monthly household income (yuan)	0–2000	71	15.1
	2001–5000	125	26.5
	5001–10000	167	35.5
	10001 and above	108	22.9
Gender of preschool children	Boy	252	53.5
	Girl	219	46.5

**TABLE 3:** Basic participant information of Sample 1 in Study 4 (*n* = 442).

Participant’s information	Classification	Frequency	Percentage (%)
Family residence	Rural	69	15.6
	Township	11	2.5
	County	129	29.2
	City	233	52.7
Educational background	Primary school	13	2.9
	Junior high school	85	19.2
	High school or technical Secondary school	97	21.9
	Junior college	124	28.1
	Undergraduate	110	24.9
	Postgraduate and above	13	2.9
Total monthly household income (yuan)	0–2000	58	13.1
	2001–5000	182	41.2
	5001–10000	149	33.7
	10001 and above	53	12
Gender of preschool children	Boy	251	56.8
	Girl	191	43.2

**TABLE 4:** Basic participant information of Sample 2 in Study 4 (*n* = 318).

Participant’s information	Classification	Frequency	Percentage (%)
Family residence	Rural	39	12.3
	Township	8	2.5
	County	115	36.2
	City	156	49.1
Educational background	Primary school	3	0.9
	Junior high school	29	9.1
	High school or technical Secondary school	64	20.1
	Junior college	79	24.8
	Undergraduate	120	37.7
	Postgraduate and above	23	7.2
Total monthly household income (yuan)	0–2000	21	6.6
	2001–5000	98	30.8
	5001–10000	107	33.6
	10001 and above	92	28.9
Gender of preschool children	Boy	178	56
	Girl	140	44

### Measures

Chinese versions were available for all questionnaire tools that were used. Each measure had previously been shown to have satisfactory reliability and validity.

### Parental Burnout Assessment

The Parental Burnout Assessment (PBA) was designed by Roskam et al. (2018), with 22 items that measured four dimensions: exhaustion in one’s parental role, contrast with previous parental self, feelings of weariness with one’s parental role, and emotional distancing from one’s children (e.g., “I can’t stand being a parent anymore”; “Apart from the usual care activities [driving, sleeping, and eating], I can’t pay more for my children”). Cheng et al. (2020) revised the Chinese version with a total of 23 items, using the 7-point scale, in which a higher score indicated a higher number of burnout symptoms. In the present study, Cronbach’s α was 0.926.

### Perceived Social Support Scale

The Chinese version of the Perceived Social Support Scale (PSSS) was revised by Jiang (2001) based on the original scale of Zimet et al. (1988; e.g., “I can freely express my opinion to my partner or group of friends”; “If I have problems, my friend/partner would help me”). The scale emphasizes an individual’s perception of social support, including the degree of support from family, friends, and others (Cohen and Matthews, 1987). The PSSS has 12 items and a Likert-type 5-point scale, from 1 (complete agreement) to 5 (complete disagreement). Higher total scores indicate higher perceived support. In the present study, Cronbach’s α was 0.925.

### University of California, Los Angeles Loneliness Scale

The Chinese version of the University of California, Los Angeles, Loneliness Scale (Wang, 1995) was revised by Wang based of the original scale of Russell et al. (1980; e.g., “How often do you feel as if nobody really understands you?”). It was based on the results from 320 college students, and an 18-item version was formed. Ten items among them were in reverse wording. Cronbach’s α was 0.895. The correlation of even-number items and odd-number items was 0.869, with an adjusted split-half reliability score of 0.925.

### Marriage Satisfaction Scale

The Marriage Satisfaction Scale was a subscale from the Marital Gratification Scale, which measures marriage quality (Schaefer and Olson, 1981). This scale contained 10 items, including five reverse-scoring items (e.g., “I don’t like my spouse’s personality and personal habits”; “I am very satisfied with the responsibilities of both spouses in the marriage”). These items were rated from 1 (indeed) to 5 (indeed not). Higher scores indicate higher marital satisfaction of parents. In the present study, Cronbach’s α was 0.869.

### Children’s Self-Efficacy Assessment Questionnaire

The Children’s Self-Efficacy Assessment Questionnaire (CSEAQ; Huang and Yang, 2015) was revised based on the General Self-Efficacy Scale (GSES; Schwarzer et al., 1997, 1999). The GSES originally consisted of 20 items (e.g., “I am confident that I can deal with any unexpected things effectively”) and was reduced to 10 items. The Chinese version with six items, scored from 1 (extremely inconsistent) to 5 (fully compliant), was used. Higher scores indicated higher self-efficacy of children. In the present study, Cronbach’s α was 0.816.

### Other Measures

Demographic items were collected, including the children’s gender and age and parents’ socioeconomic status and education level.

### Data Analyses

During the first stage of developing the present scale, an extensive literature review and interviews were conducted to generate the scale’s items. For the literature review, the keywords “parental involvement and support,” “parental involvement and support of preschool children,” and “family support” were searched in both Chinese and English literature databases. Parents and grandparents were asked five questions about parental support and involvement, which were compiled by the authors and experts in preschool education. For example, “What kind of parental involvement and support do you provide to your child?” “Which aspect of parental involvement and support do you want to improve the most?” “Is there any pressure on your family to support your child? What are the pressures?” By collating the literature and interview data, we found that parental involvement and support for preschool children were primarily reflected in daily life, material conditions, emotions, education, quality time, mental health, play, and entertainment.

In Study 2 and Study 3, two rounds of item analysis and Exploratory Factor Analysis were conducted to examine the appropriateness of the items and ensure validity of the scale. Through correlation analysis and difference testing between high and low groups, inappropriate items were eliminated, and Principal Components Analysis and maximum variance were used to analyze the data. Common factors were extracted to explore dimensions and structural validity of the scale, and dimensions that were formed by common factors were named.

In Study 4, Confirmatory Factor Analysis and correlation analysis were conducted for the data from Sample 1 to test constructive validity and internal validity of the CPISSPC. To test concurrent validity of the CPISSPC, the data from Sample 2 were used to analyze correlations between parental involvement and support, parental burnout, perceived social support, loneliness, marriage satisfaction, and children’s self-efficacy.

These Exploratory Factor Analyses and correlation analyses were performed using SPSS 22.0 software. AMOS 24.0 software was used for the Confirmatory Factor Analysis.

## Results

### Study 1. Initial Item Development

The first author of the present study created an initial pool of 58 items based on the literature search and interview results, which were reviewed by other authors. Several of the items were revised. Three experts in preschool education and five parents of preschool children were then invited to evaluate content validity of the scale and revise the items according to the following criteria: (1) items with unclear and inaccurate language expressions, (2) repetitions of other items, (3) items that were difficult for parents of preschool children to understand, and (4) items that did not reflect well the content of family support for preschool children. Finally, experts and parents of preschoolers were asked to review the revised items again. Through the literature review and interviews, we preliminarily determined the content of parental involvement and support for preschool children and compiled 58 items. We then invited experts to modify the content and language of the items. We then preliminarily determined the initial scale.

### Study 2. Item Analysis and Exploratory Factor Analysis of the First Round

The item analysis included calculating the critical ratio (CR) and performing the item homogeneity test. The CR was used to calculate the difference of each item for the 27% highest scores group versus the 27% lowest scores group. Items were deleted if the CR was less than 3. Item-total statistics were used to measure content homogeneity between items, with a value greater than 0.3 indicating that the item correlated well with the total scale (Table 5). A total of 47 items survived the two criteria of the item analysis.

**TABLE 5:** Item analysis results in Study 2 (*n* = 447).

Item	Critical ratio	Correlation coefficient	Statistical significance
1	10.122	0.457**	0.000**
2	8.211	0.458**	0.000**
3	5.182	0.279**	0.000**
4	12.387	0.517**	0.000**
5	6.299	0.306**	0.000**
6	6.668	0.374**	0.000**
7	6.697	0.352**	0.000**
8	7.525	0.323**	0.000**
9	7.069	0.402**	0.000**
10	7.616	0.359**	0.000**
11	8.973	0.391**	0.000**
12	3.380	0.197**	0.001**
13	2.353	0.16**	0.019*
14	−3.517	−0.191**	0.001**
15	7.797	0.399**	0.000**
16	7.342	0.369**	0.000**
17	5.847	0.279**	0.000**
18	8.620	0.484**	0.000**
19	4.577	0.316**	0.000**
20	9.912	0.508**	0.000**
21	12.999	0.61**	0.000**
22	13.117	0.653**	0.000**
23	7.005	0.318**	0.000**
24	2.705	0.138**	0.007**
25	5.807	0.37**	0.000**
26	9.038	0.565**	0.000**
27	4.406	0.226**	0.000**
28	7.138	0.33**	0.000**
29	8.942	0.407**	0.000**
30	6.298	0.324**	0.000**
31	12.655	0.529**	0.000**
32	5.313	0.212**	0.000**
33	9.772	0.514**	0.000**
34	10.215	0.55**	0.000**
35	14.306	0.685**	0.000**
36	9.805	0.524**	0.000**
37	4.667	0.21**	0.000**
38	8.138	0.405**	0.000**
39	11.691	0.584**	0.000**
40	11.195	0.555**	0.000**
41	8.078	0.362**	0.000**
42	9.190	0.497**	0.000**
43	8.225	0.551**	0.000**
44	7.517	0.509**	0.000**
45	9.151	0.55**	0.000**
46	8.533	0.456**	0.000**
47	10.094	0.529**	0.000**
48	9.989	0.593**	0.000**
49	11.134	0.587**	0.000**
50	14.549	0.678**	0.000**
51	12.027	0.66**	0.000**
52	9.832	0.459**	0.000**
53	6.066	0.308**	0.000**
54	10.946	0.501**	0.000**
55	4.225	0.183**	0.000**
56	8.273	0.463**	0.000**
57	5.257	0.241**	0.000**
58	9.931	0.468**	0.000**

*p < 0.05, **p < 0.01.

Exploratory Factor Analysis of the 47 items was then conducted using Principal Components Analysis and maximum variance. The results showed that the Kaiser–Meyer–Olkin (KMO) value was 0.923, which is greater than 0.6, and the Bartlett test value was 8242.141 (*df* = 1081, *p* < 0.001), indicating that there were common factors in the correlation coefficient matrix and that the scale items possessed high commonality, which was suitable for factor analysis.

Six common factors with eigenvalues greater than 1 were extracted, and inappropriate items were deleted according to the following two conditions: loading value less than 0.4 and cross-loading value on several common factors. As a consequence, 17 items were deleted. The cumulative explanatory variance of the six common factors was 55.6% (Table 6). Next, the six dimensions were named educational method (Factor 1), psychological concern (Factor 2), life care (Factor 3), understanding of play (Factor 4), educational behavior (Factor 5), and educational concept (Factor 6).

**TABLE 6:** Results of component matrix after rotation in Study 2 (*n* = 447).

Item	Factor 1	Factor 2	Factor 3	Factor 4	Factor 5	Factor 6
43	0.783					
44	0.729					
45	0.706					
40	0.676					
42	0.668					
39	0.645					
48	0.63					
46	0.468					
47	0.456					
20		0.669				
33		0.652				
21		0.562				
34		0.557				
49		0.518				
50		0.51				
56		0.501				
22		0.495				
4			0.716			
2			0.711			
1			0.656			
6			0.545			
7			0.48			
52				0.723		
54				0.654		
29					0.785	
23					0.595	
36					0.465	
11						0.733
9						0.649
10						0.621
Eigenvalue	9.74	2.208	1.637	1.29	1.242	1.111
Contribution Rate	15.85%	11.91%	8.67%	6.77%	6.34%	6.05%
Cumulative Contribution Rate	15.85%	27.76%	36.43%	43.19%	49.53%	55.58%

### Study 3. Item Analysis and Exploratory Factor Analysis of the Second Round

In this round of item analysis, all items and total scores were significantly correlated at a level of 0.01 (Table 7). Specifically, 26 items had a correlation coefficient greater than 0.5, accounting for 74.3% of the total number of items. The correlation coefficients of the six items were greater than 0.4, accounting for 17.1% of the total items, and the correlation coefficients of the three items were greater than 0.3, accounting for 8.6% of the total items.

**TABLE 7:** Item analysis results in Study 3 (*n* = 471).

Item	Critical ratio	Correlation coefficient	Statistical significance
1	−8.954	0.434**	0.000**
2	−10.413	0.416**	0.000**
3	−16.021	0.647**	0.000**
4	−14.331	0.543**	0.000**
5	−9.261	0.443**	0.000**
6	−9.713	0.464**	0.000**
7	−7.603	0.328**	0.000**
8	−12.496	0.574**	0.000**
9	−9.665	0.424**	0.000**
10	−4.989	0.304**	0.000**
11	−13.35	0.489**	0.000**
12	−13.584	0.546**	0.000**
13	−16.775	0.657**	0.000**
14	−11.744	0.536**	0.000**
15	−16.349	0.617**	0.000**
16	−11.79	0.575**	0.000**
17	−10.82	0.518**	0.000**
18	−15.072	0.595**	0.000**
19	−18.368	0.695**	0.000**
20	−14.792	0.539**	0.000**
21	−16.61	0.579**	0.000**
22	−14.799	0.69**	0.000**
23	−8.426	0.372**	0.000**
24	−16.908	0.667**	0.000**
25	−19.129	0.68**	0.000**
26	−13.546	0.564**	0.000**
27	−15.333	0.642**	0.000**
28	−16.219	0.655**	0.000**
29	−14.813	0.64**	0.000**
30	−11.802	0.575**	0.000**
31	−13.355	0.546**	0.000**
32	−17.955	0.675**	0.000**
33	−14.303	0.621**	0.000**
34	−16.603	0.713**	0.000**
35	−20.147	0.711**	0.000**

**p < 0.01.

To form a more stable dimension structure of the scale, Principal Components Analysis was performed on 35 items. The KMO value of the Exploratory Factor Analysis in the second round of analysis was 0.895 (i.e., greater than 0.6). The Bartlett test value was 3029.383 (*df* = 153, *p* < 0.001), thus demonstrating its suitability for factor analysis. According to the standard of deleting items that were used in the first round of testing, 18 items were retained.

Finally, four stable dimensions were formed, with four to six items in each dimension (Table 8). The four factors were named psychological support (Factor 1), play support (Factor 2), educational support (Factor 3), and life support (Factor 4). The items and dimensions of the scale were then determined, and exploratory analysis was completed.

**TABLE 8:** Results of component matrix after rotation in Study 3 (*n* = 471).

Item	Factor 1	Factor 2	Factor 3	Factor 4
29	0.744			
25	0.717			
22	0.708			
24	0.687			
28	0.68			
26	0.673			
21		0.829		
32		0.786		
31		0.686		
33		0.551		
9			0.744	
11			0.737	
12			0.727	
20			0.616	
1				0.715
10				0.707
6				0.572
4				0.534
Eigenvalue	6.249	1.56	1.398	1.229
Contribution Rate	19.612%	13.782%	13.468%	11.118%
Cumulative Contribution Rate	19.612%	33.394%	46.863%	57.981%

### Study 4. Examination of Reliability and Validity

#### Reliability Analysis

Cronbach’s α of the whole CPISSPC scale and each dimension was between 0.649 and 0.88, which fell within an acceptable range. This indicates that the CPISSPC had good reliability.

#### Confirmatory Factor Analysis

Based on the four-factor theoretical model, the factor structure of the CPISSPC was analyzed by the maximum likelihood method using Amos 24.0 software. The factor loading of each index on the potential variables was greater than 0.45 (Table 9). The results of the model fitting indices showed χ*^2^* = 363.562, *df* = 129, *p* < 0.001, CFI = 0.912, RMSEA = 0.064, SRMR = 0.057, χ*^2^*/*df* = 2.818. Therefore, the revised four-factor model of preschool children’s parental involvement and support scale had good structural validity.

**TABLE 9:** Results of Confirmatory Factor Analysis (*n* = 442).

Item	PS	ES	PLS	LS
10. I am a friend to my child.	0.684			
11. I communicate with my child daily about the day’s activities.	0.702			
12. I trust my child. I do not ridicule him/her just because he/she is young.	0.706			
13. I reassure my child that he/she is loved and wanted.	0.648			
14. I often praise my child.	0.659			
15. I supervise and participate in play with my child.	0.654			
4. I encourage my child to play with other children.		0.51		
6. I provide opportunities for my child to self-feed and self-dress.		0.755		
7. I encourage my child to use polite expressions in appropriate situations, such as “thank you,” “sorry,” and “welcome.”		0.713		
8. I teach my child that he/she should not fight for toys; rather share or play in turns.		0.762		
9. Play is a way of learning.			0.488	
16. It is a basic right of children to think that play is beneficial.			0.615	
17. I share thoughts and experiences with my child after play.			0.781	
18. There is playing space for my child at home.			0.567	
1. I prepare a balanced diet for my child.				0.473
2. I prioritize quality when buying clothes for my child.				0.539
3. My child has his/her own room or bed.				0.52
5. I buy new clothes for my child regularly.				0.684

*PS, psychological support; ES, educational support; PLS, play support; LS, life support.*

#### Internal Validity

SPSS 21.0 software was used to conduct the related analysis. The correlation coefficient between the CPISSPC for each dimension and total score of the questionnaire was 0.659–0.849 (*p* < 0.01). The correlation coefficient between scores for each dimension was 0.318–0.594 (*p* < 0.01). The correlation coefficient between the score for each dimension and the total score was greater than the correlation coefficient between dimensions, and this difference reached statistical significance, indicating that each dimension had a certain degree of independence (Table 10).

**TABLE 10:** Correlation analysis results of parental involvement and support scale for preschool children (*n* = 318).

	PS	ES	PLS	LS	FIS
PS	1				
ES	0.455**	1			
PLS	0.594**	0.484**	1		
LS	0.353**	0.361**	0.318**	1	
FIS	0.849**	0.693**	0.799**	0.659**	1

PS, psychological support; ES, educational support; PLS, play support; LS, life support; FIS, family internal support, **p < 0.01.

#### Criterion Correlation Validity

The results showed that the correlation coefficient between the CPISSPC total score and the total score of each criterion tool ranged from 0.299 to 0.39 (*p* < 0.01). The correlation coefficient between the total score of each dimension of the CPISSPC and total score of each criterion tool ranged from 0.159 to 0.373 (*p* < 0.01; Table 7). The dimensions and total scale significantly correlated with the five criterion scales, indicating that the scale had good criterion validity (Table 11).

**TABLE 11:** Criterion validity results of preschool children’s parental involvement and support and school standard variables (*n* = 318).

	PBA	PSSS	LO	MG	SE
	α = 0.926	α = 0.925	α = 0.895	α = 0.869	α = 0.816
PS	−0.303**	0.329**	−0.373**	0.247**	0.372**
ES	−0.173**	0.291**	−0.293**	0.200**	0.236**
PLS	−0.205**	0.353**	−0.324**	0.230**	0.267**
LS	−0.19**	0.159**	−0.171**	0.222**	0.221**
FIS	−0.299**	0.376**	−0.39**	0.3**	0.375**

PS, psychological support; ES, educational support; PLS, play support; LS, life support; FIS, family internal support; PBA, parental burnout assessment; PSSS, Perceived Social Support Scale; LO, loneliness; MG, marital gratification; SE, self-efficacy **p < 0.01.

The reliability analysis, Confirmatory Factor Analysis, internal validity, and criterion correlation validity demonstrated that the CPISSPC had good reliability and validity.

## Discussion and Conclusion

The main purpose of the present study was to develop and validate a scale to measure parental involvement and support for preschoolers in China. The results suggested that the psychometric properties of the CPISSPC were acceptable. Cronbach’s α for the scale was 0.88. The CPISSPC includes four factors: life support, psychological support, educational support, and play support. The Exploratory Factor Analysis results indicated that the cumulative contribution rate of the four factors explained 57.981% of the variance. The Confirmatory Factor Analysis results indicated that the life support, psychological support, educational support, and play support factor loadings were all above 0.40. No cross loadings were identified. Concurrent validity indicated that parental involvement and support positively correlated with social support, self-efficacy, and marriage quality scores and negatively correlated with parents’ levels of burnout and loneliness. To our knowledge, this scale is the first to comprehensively measure parental involvement and support for preschoolers in the Chinese context.

### Construct Validity of the Chinese Parental Involvement and Support Scale for Preschool Children

Construct validity was investigated in two aspects. The first was to explore factors that affected parental involvement and support from aspects of the parents. The second was to explore the impact of parental involvement support on children from aspects of the children. The results showed that a high level of marital gratification and perceived social support positively correlated with parental involvement support. In contrast, parents’ high levels of loneliness and burnout could hinder parental involvement and support. These results indicated that if we want to encourage parents to have more involvement and provide more support for their children, then we need to pay attention to reducing their burnout and loneliness, helping them improve their marital satisfaction, and providing more social support. We also found that when parents devoted themselves more to raising their children, their children’s self-efficacy improved. This result provided further evidence of the positive impact of parental involvement and support on children’s development.

### Four-Factor Model of the Chinese Parental Involvement and Support Scale for Preschool Children

Life support or material support is the basis for the development of preschool children. Surprisingly, however, no scale has been previously developed for preschool children that measures life support that is provided by children’s families. In the CPISSPC, the life support factor consists of four items: balanced nutrition, providing children with their own bed or room, buying clothes regularly, and prioritizing the quality of clothes. A previous study explored the relationship between dietary patterns and cognitive development and found higher full-scale IQ at 4 years of age in children who consumed more fruit, vegetables, and food overall (Gale et al., 2009). For preschool children, the rate of physical development is rapid (Tran et al., 2019), so clothing should be selected with “short-term” use in mind. Therefore, we determined that life support or material support should be included in the present parental involvement and support scale.

Psychological support was an important factor in the CPISSPC. Previous scales about parenting also included some aspects of psychological support. However, compared with the previous scales, the CPISSPC has some advantages. For example, the CECPAQ has a support dimension (13 items) that refers to the extent to which parents are attuned, supportive, and acquiescent to their child’s needs and demands (Dong et al., 2021). In the CPISSPC, psychological support includes six items: treat the child in a friendly manner, communicate with the child about daily life, trust the child, praise the child, express love toward the child, and accompany the child while playing. In our opinion, the CECPAQ mainly focuses on children’s emotional needs, whereas the CPISSPC covers a wider range.

The educational support factor in the CPISSPC consists of encouraging the child to play with peers, independence, politeness, and conflict resolution. Playing with peers, independence, and politeness have been included in previous parenting scales. However, these previous parenting scales failed to include the conflict resolution item. For example, the “play with other children” item in the present scale is very similar to the “I regularly let my child play with other children” item in the CECPAQ, which belongs to the “exposure” dimension (Dong et al., 2021). The CRPR also has items on encouraging independence (“If my child gets into trouble, I expect him or her to handle the problem mostly by himself or herself”; Chen et al., 1998). In the CPPM (Wu et al., 2002), the “encouragement of modesty” dimension (Wu et al., 2002) is similar to the politeness item in the CPISSPC. The conflict resolution item is not seen in other scales. Considering that children often have conflicts over toys, parents should teach their children to resolve conflicts with peers.

Play is key to preschool children’s learning, development, confidence, and wellbeing (Yogman et al., 2018). Playing with toys is an important form of play. Parents generally view toys as important for children’s development. Nonetheless, the majority of preschool children scales do not measure the content of toys. Only the CECPAQ has a four-item subscale that measures the extent to which parents engage with their child while playing with toys. These four items only involve four kinds of toys. In the CPISSPC, the play dimension also includes four items: two items that assess parents’ attitudes about toys, one item that evaluates whether parents provide space for children to play with toys, and one item that asks parents if they exchange ideas and experiences about toys after their children play with toys. Unlike the toys subscale in the CECPAQ, which only focuses on toy types, the play dimension of the CPISSPC covers a wider range.

## Strengths and Limitations

The CPISSPC has several advantages over existing scales that measure parental involvement and support. First, the CPISSPC has four relatively distinct and comprehensive dimensions that better represent the content of parental involvement and support for preschool children. Second, in contrast to parenting practice scales that have been translated and revised from abroad, the CPISSPC is based on interviews directly with Chinese parents. Therefore, the content of the CPISSPC more aligns with the actual situation of parenting practices in China. Third, the CPISSPC has only 18 items. Therefore, it can be completed in a relatively short period of time and will likely be useful for studies that administer several scales.

The present study has limitations. First, different countries and areas have great differences in social development, economic level, cultural tradition, and education concepts, among others. The psychometric properties of the CPISSPC were assessed only among parents from some cities in China, which may not be representative of other countries. Future studies should include parents from other countries and areas to compare similarities and differences in their levels of parental involvement and support. However, the selection of participants for the assessment of our scale was relatively strict, the scale development process was standardized. Moreover, the CPISSPC has good psychometric properties and can be used as a tool to measure parental involvement and support for preschool children. If other countries and areas want to use the CPISSPC, then the scale could be adapted or localized. Second, when evaluating the concurrent validity of the CPISSPC, we only explored correlations among factors about parents and parental involvement and support. We did not explore the influence of specific levels of parental involvement and support on children’s development. Future studies should explore how parents’ levels of involvement and support affect children’s development.

## Conclusion

The CPISSPC is a comprehensive and valid measure of parental involvement and support for preschool children. The present study suggests that the scale has a four-factor structure that consists of psychological support, educational support, play support, and life support. The scale has good stability and consistency and can be used as an effective tool to evaluate the level of parental involvement and support for preschool children in China.

## Data Availability Statement

The raw data supporting the conclusions of this article will be made available by the authors, without undue reservation.

## Ethics Statement

The studies involving human participants were reviewed and approved by Sub Committee of Biomedical Science Ethics, Henan University. The patients/participants provided their written informed consent to participate in this study. Written informed consent was obtained from the individual(s) for the publication of any potentially identifiable images or data included in this article.

## Author Contributions

YY and XZ were responsible for revising the manuscript. YZ and WR were responsible for the data analysis and writing of the manuscript. All authors contributed to the article and approved the submitted version.

## Conflict of Interest

The authors declare that the research was conducted in the absence of any commercial or financial relationships that could be construed as a potential conflict of interest.

## Publisher’s Note

All claims expressed in this article are solely those of the authors and do not necessarily represent those of their affiliated organizations, or those of the publisher, the editors and the reviewers. Any product that may be evaluated in this article, or claim that may be made by its manufacturer, is not guaranteed or endorsed by the publisher.
